# Review of the subgenus Plumiger of *Myrmecophyes*, with description of a new species (Heteroptera, Miridae, Halticini)

**DOI:** 10.3897/zookeys.796.21877

**Published:** 2018-11-15

**Authors:** Fedor V. Konstantinov, Nikolay Simov

**Affiliations:** 1 Department of Entomology, Faculty of Biology, St. Petersburg State University, Universitetskaya nab. 7/9, St. Petersburg 199034, Russia St. Petersburg State University St. Petersburg Russia; 2 Zoological Institute, Russian Academy of Sciences, Universitetskaya nab. 1, St. Petersburg 199034, Russia Zoological Institute, Russian Academy of Sciences St. Petersburg Russia; 3 National Museum of Natural History, Bulgarian Academy of Sciences, 1 Tsar Osvoboditel Blvd., 1000 Sofia, Bulgaria National Museum of Natural History, Bulgarian Academy of Sciences Sofia Bulgaria

**Keywords:** Alpine meadows, Caucasus, diagnosis, female genitalia, key to species, male genitalia, systematics

## Abstract

The Caucasian subgenus Plumiger Horváth, 1927 of the halticine genus *Myrmecophyes* Fieber, 1870 is revised. A key, updated diagnoses, and data on distribution are given for the subgenus and its four species, including *M.tomi***sp. n.** (Georgia and Dagestan), and the previously unknown male of *M.armeniacus* Drapolyuk, 1989. Illustrations of the male and female genitalia, photographs of the dorsal habitus, and SEM micrographs of selected structures are provided for all species of the subgenus.

## Introduction

*Myrmecophyes* Fieber, 1870 is a distinctive and strikingly myrmecomorphic halticine genus comprising 30 currently recognized species (Schuh 2002–2013, Tatarnic and Cassis 2012, Konstantinov et al. 2013). All *Myrmecophyes* spp. are brachypterous, restricted to relatively small distributional areas and inhabit montane grasslands, except *M.alboornatus*, which spans a large area in Europe and Siberia, reaching Kazakhstan, Mongolia, and northern China in the south. The genus is most speciose in the Central Asian and Caucasian mountains. Species found elsewhere are *M.gallicus* Wagner, 1976 in the Pyrenees; *M.latus* Wagner, 1975 and *M.montenegrinus* Wagner, 1976 described from Western Balkan Mountains; and *M.oregonensis* Schuh & Lattin, 1980, the only North American species, found in Oregon at altitudes from 1500 to 1700 m.

Horváth (1927) described the subgenus Plumiger within *Myrmecophyes* to accommodate *M.heterocerus* Horváth, 1927. His diagnosis of the monotypic subgenus was based on the modified first two antennal segments in males. Drapolyuk (1989) published a review of the Caucasian *Myrmecophyes* and described two more species of the subgenus.

Recent collecting by the authors from the Armenian Highlands produced a substantial number of *Myrmecophyes* spp., including long series of Myrmecophyes (Plumiger) armeniacus Drapolyuk, 1989, originally described from two females. Subsequent examination of holdings from the Zoological Institute, Russian Academy of Sciences (ZISP), led to the discovery of a new species belonging to the same subgenus. Thus we recognize four species within *Plumiger*, all restricted to the Caucasus. A phylogenetic analysis of the genus is needed to clarify the status of the subgenus. Pending such a study, which might reveal interesting biogeographic patterns, we are certain that the group is sufficiently distinct to warrant recognition at the subgeneric level. The monophyly of *Plumiger* is corroborated by characters presented in our diagnosis that are not shared by other species of *Myrmecophyes*.

The present paper provides the description of a new species, a key to males and females, a revised diagnosis, and a redescription of the subgenus. A diagnosis, description, measurements, distributional information, a dorsal habitus photograph, and illustrations of male and female genitalia are given for each species of the subgenus.

We are delighted to dedicate this paper to our eminent colleague Dr. Thomas J. Henry on the occasion of his 70^th^ birthday. During a long and distinguished career at the USDA, c/o Smithsonian National Museum of Natural History, Tom Henry has made singular contributions to our knowledge of the Heteroptera worldwide. Despite his many duties, Tom always manages to find time to help his colleagues in their studies and to provide an energetic and supportive environment to all researchers working with the USNM Heteroptera collection.

## Materials and methods

Slightly more than 600 specimens were examined for this study. All specimens are retained in the Zoological Institute, St. Petersburg, Russia (**ZISP**) and the National Museum of Natural History, Sofia, Bulgaria (**SOFM**). The holotype of *Myrmecophyestomi* sp. n. is deposited in the Heteroptera collection of the Zoological Institute in St. Petersburg. All ZISP specimens were associated with bar code labels (“unique specimen identifiers” or “USIs”), which were printed as a matrix code label that also provides an alphanumeric string, e.g., AMNH_PBI 00343231. USI numbers explicitly identify particular specimens and are listed for each species in the “Material examined” section. Additional specimen information for a selected species, including additional figures not included in this paper, can be obtained from the Heteroptera Species Pages (http://research.amnh.org/pbi/heteropteraspeciespage/), which assemble available data from a specimen database. Geo-reference data for each locality were obtained from gazetteers, atlases, handheld GPS and other sources. The distributional maps were created using QGIS 2.18.4 software.

Unless otherwise stated, all measurements are in millimeters. Measurements shown in Table 1 include body length, clypeus to apex of wing pad length, head and pronotum length and width, interocular distance, length of hind tibia, and antennal segments I and II.

**Table 1.** Measurements (mm). Abbreviations: Clyp-Wing – distance between apex of clypeus and apex of wing pad in dorsal view, Head Length – distance between apex of clypeus and the highest point of vertex, AntSeg1 – AntSeg2 – length of antennal segments I and II, InterOcDi – width of vertex between inner margins of eyes in dorsal view.

Species	Length								Width		
		Body	Clyp-Wing	Head	Pronotum	Tibia3	AntSeg1	AntSeg2	Head	InterOcDi	Pronotum
*** Myrmecophyes armeniacus ***											
♂♂ (n = 5)	**Mean**	**3.46**	**2.07**	**0.55**	**0.65**	**3.27**	**0.48**	**0.67**	**1.15**	**0.56**	**0.85**
	**SD**	0.11	0.07	0.03	0.02	0.11	0.02	0.03	0.02	0.02	0.03
	**Range**	0.25	0.16	0.09	0.04	0.28	0.04	0.07	0.04	0.04	0.07
	**Min**	3.40	2.02	0.50	0.64	3.08	0.46	0.64	1.13	0.53	0.81
	**Max**	3.65	2.18	0.58	0.67	3.36	0.50	0.71	1.17	0.57	0.88
♀♀ (n = 5)	**Mean**	**3.99**	**2.17**	**0.52**	**0.65**	**3.02**	**0.51**	**1.00**	**1.20**	**0.60**	**0.89**
	**SD**	0.11	0.11	0.05	0.02	0.11	0.02	0.02	0.04	0.03	0.03
	**Range**	0.25	0.28	0.11	0.04	0.28	0.05	0.04	0.07	0.07	0.07
	**Min**	3.86	2.05	0.46	0.64	2.90	0.48	0.99	1.17	0.57	0.85
	**Max**	4.11	2.34	0.57	0.67	3.19	0.53	1.03	1.24	0.64	0.92
*** Myrmecophyes heterocerus ***											
♂♂ (n = 5)	**Mean**	**2.97**	**1.71**	**0.48**	**0.52**	**1.88**	**0.42**	**0.53**	**0.99**	**0.46**	**0.71**
	**SD**	0.16	0.12	0.06	0.02	1.07	0.00	0.03	0.02	0.01	0.03
	**Range**	0.42	0.32	0.14	0.04	2.58	0.00	0.07	0.05	0.04	0.07
	**Min**	2.76	1.52	0.42	0.50	0.00	0.42	0.50	0.97	0.44	0.67
	**Max**	3.19	1.84	0.57	0.53	2.58	0.42	0.57	1.03	0.48	0.74
♀♀ (n =5)	**Mean**	**3.37**	**1.90**	**0.53**	**0.57**	**2.39**	**0.48**	**0.88**	**1.11**	**0.56**	**0.84**
	**SD**	0.38	0.10	0.07	0.03	0.10	0.03	0.05	0.04	0.03	0.03
	**Range**	0.92	0.21	0.18	0.07	0.25	0.07	0.11	0.09	0.05	0.07
	**Min**	2.90	1.81	0.46	0.53	2.30	0.46	0.81	1.08	0.53	0.81
	**Max**	3.82	2.02	0.64	0.60	2.55	0.53	0.92	1.17	0.58	0.88
*** Myrmecophyes nasutus ***											
♂♂ (n = 2)	**Min**	3.60	2.05	0.46	0.60	3.36	0.85	1.56	1.24	0.64	0.85
	**Max**	3.75	2.30	0.57	0.64	3.54	0.85	1.59	1.26	0.65	0.88
♀♀ (n =5)	**Mean**	**4.30**	**2.19**	**0.59**	**0.67**	**3.25**	**0.64**	**1.48**	**1.30**	**0.68**	**0.99**
	**SD**	0.11	0.05	0.03	0.01	0.08	0.02	0.07	0.03	0.03	0.03
	**Range**	0.28	0.11	0.07	0.02	0.21	0.04	0.18	0.09	0.07	0.07
	**Min**	4.18	2.12	0.57	0.65	3.12	0.64	1.42	1.26	0.64	0.96
	**Max**	4.46	2.23	0.64	0.67	3.33	0.67	1.59	1.35	0.71	1.03
***Myrmecophyestomi* sp. n.**											
♂♂ (n = 1)		3.40	1.88	0.46	0.64	3.19	0.57	0.99	1.13	0.57	0.85
♀♀ (n =5)	**Mean**	**3.33**	**1.84**	**0.52**	**0.57**	**2.48**	**0.46**	**0.86**	**1.10**	**0.56**	**0.81**
	**SD**	0.16	0.10	0.05	0.03	0.20	0.04	0.04	0.04	0.02	0.04
	**Range**	0.42	0.25	0.11	0.07	0.46	0.07	0.11	0.11	0.05	0.11
	**Min**	3.12	1.70	0.46	0.53	2.30	0.42	0.81	1.03	0.53	0.74
	**Max**	3.54	1.95	0.57	0.60	2.76	0.50	0.92	1.13	0.58	0.85

Observations, measurements, and digital dorsal color images were made with a Nikon SMZ 1500 stereomicroscope equipped with Nikon D700 digital SLR camera. Images of the genitalic structures were taken with a Leica DM2500 microscope equipped with Leica DFC 450 digital camera. Partially focused images of each specimen or structure were stacked using the Helicon Focus 6.2.2 software. The terminology used for genitalia follows Konstantinov (2003) for males and Schwartz (2011) for females.

**Figures 1–4.**
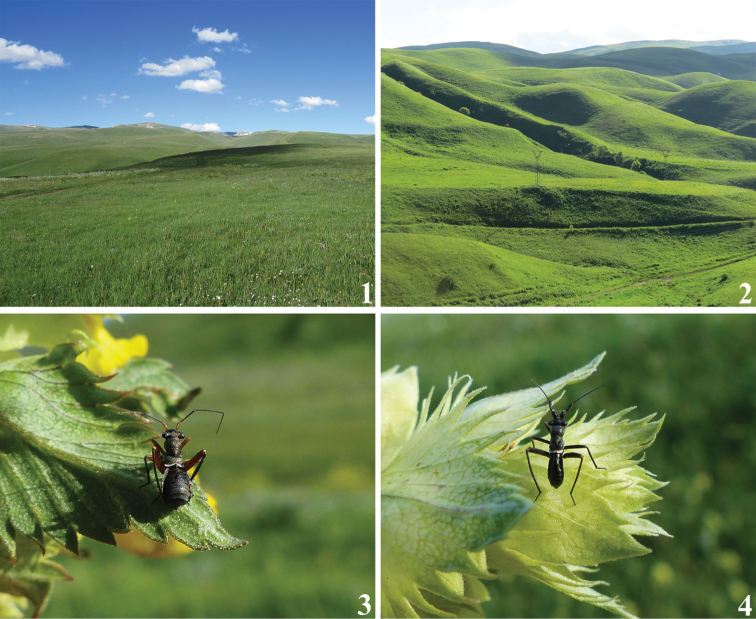
Habitats and habitus images of live individuals of Myrmecophyes (Plumiger) species. **1, 2** Typical habitats of Armenian Myrmecophyes (Plumiger) species **3, 4***Myrmecophyesarmeniacus* Drapolyuk, 1989 **3** female **4** male.

## Taxonomy

### Genus *Myrmecophyes* Fieber, 1870

#### Subgenus Plumiger

Taxon classificationAnimaliaHemipteraMiridae

Horváth, 1927

Plumiger Horváth, 1927: 189 (new subgenus). Type species by monotypy: Myrmecophyesheterocerus Horváth, 1927.

##### Diagnosis.

Antenna sexually dimorphic (Figs 5–13), female filiform, male with first two segments modified and clothed ventrally with dense spatulate whitish scales (Figs 20, 21), segment I distinctly swollen, segment II curved and flattened apically; antennal segment III distinctly longer than other segments in both sexes; endosoma of aedeagus without sclerites, composed of several minutely dentate lobes, entirely membranous (Figs 37, 39) or slightly sclerotized (Figs 47, 49).

##### Remarks.

All species of the subgenus Myrmecophyes differ from *Plumiger* in having filiform (rather than sexually dimorphic) antennae and two or three variously shaped and typically large sclerites of the endosoma (Bykov 1971: figs 48–96, Schuh and Lattin 1980: fig. 10, Drapolyuk 1989: figs 7–10, Drapolyuk and Kerzhner 2000: figs 14, 22, Konstantinov et al. 2013: fig. 3). Antennal segments in males and females of *Myrmecophyes* spp. are straight, rod-shaped, and thin, with the second segment the longest and only slightly thinner than the first.

##### Redescription.

**Figures 5–13.**
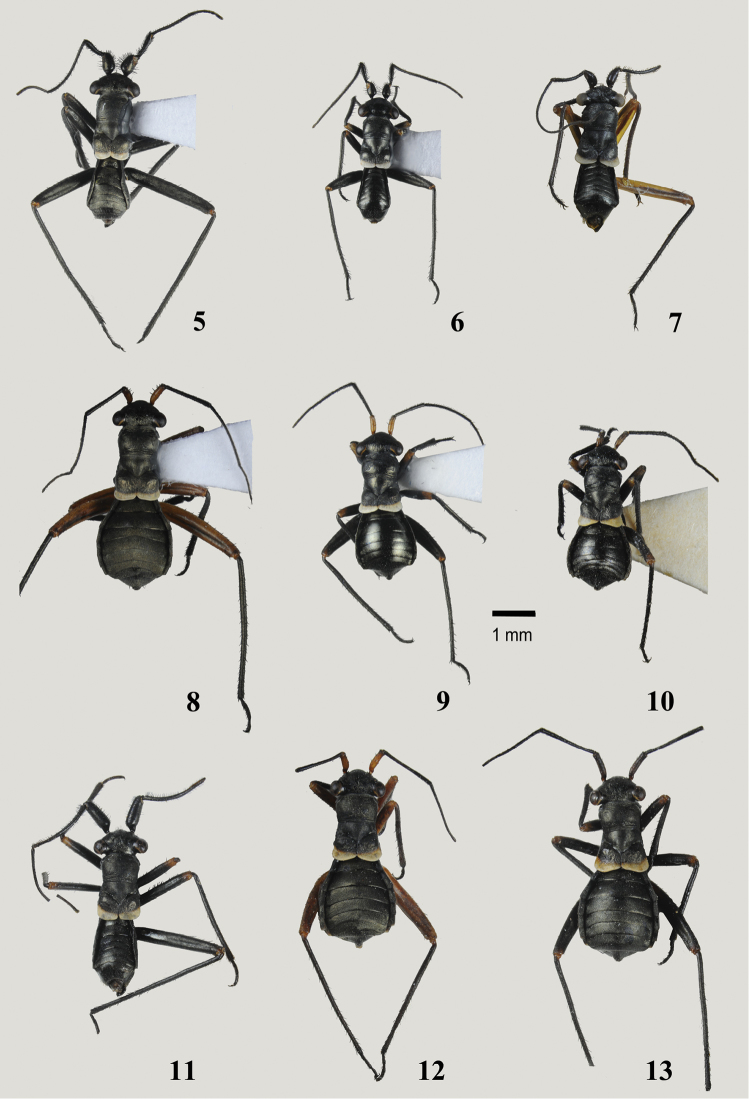
Dorsal habitus of Myrmecophyes (Plumiger) species. **5, 8***Myrmecophyesarmeniacus* Drapolyuk, 1989 **5** male **8** female **6, 9***Myrmecophyesheterocerus* Horváth, 1927 **6** male **9** female **7, 10***Myrmecophyestomi* sp. n. **7** male **10** female **11–13***Myrmecophyesnasutus* Drapolyuk, 1989 **11** male **12** pale female **13** dark female.

***Male***. Total body length 2.8–3.8, brachypterous and distinctly antlike. *Coloration* (Figs 5–7): Dorsum, thoracic pleura, and venter uniformly dark brown to black, with narrow, contrastingly whitish stripe along apex of wing pad forming transverse band; antenna uniformly black, sometimes with chestnut brown segment I; femora black, sometimes with pale brown apices, rarely almost uniformly pale brown, tibiae and tarsi dark brown. *Surface and Vestiture*: Dorsum and venter rugose, pronotum with fine transverse wrinkles along anterior and posterior margin; abdomen smoother than thoracic dorsum, sometimes shining. Dorsum, venter and legs with very short and thin, reclining, pale simple setae, scarce on head, thorax and femora, more dense on abdomen and tibiae. Head with several spinelike setae on vertex posteriorly and on frons between eye and antennal fossa, apex of frons and base of clypeus with very dense, thin and exceptionally long straight whitish setae; antennal segments I and II with numerous black spinelike setae on dorsal surfaces and with dense spatulate white scales on ventral surfaces except basal one-fourth; segments III and IV with mixture of relatively scarce erect spinelike setae shorter than those on previous segments and dense semierect pale simple setae. Pronotum with two black spinelike setae on anterorlateral corners, sometimes with additional setae along anterior and posterior margins; fore coxa with row of black spinelike setae on anterior surface; femora typically with one or two incomplete rows of stiff spinelike setae along dorsal margin and several apical spines dorsally and ventrally; tibia with scattered black spines. Genital capsule with relatively long, thin, simple setae apically and near parameres. *Structure*: Head: Large, distinctly vertical, twice as high as length or height of pronotum (Figure 15); frons flat; vertex broad and almost flat, in frontal view at about same level as dorsal margin of eyes; eyes relatively small, not stylate, projecting well beyond lateral margins of pronotum; antennal fossa below ventral margin of eye; antennal segment I distinctly swollen, barrel-shaped, 2.5–3.5 × as wide as segment II, 1.5–2.4 × as long as wide (Figure 18); segment II gradually curved and somewhat flattened at apex, segments III-IV thin, segment III distinctly or at least slightly longer than any other antennal segment; labium stout, always surpassing hind coxa and reaching basal abdominal segments. Thorax: Pronotum with anterior margin slightly convex, flattened and weakly reflexed, lateral margins strongly convex and smoothly rounded, posterior margin flattened, straight or slightly concave; calli not delimited; exposed part of mesonotum large, slightly shorter than pronotal length, distinctly convex, gibbous in lateral view; scutellum not delimited from mesonotum; hemelytron reduced to undifferentiated, broadly rounded wing pad reaching extreme base of abdomen, somewhat convex in basal two-thirds, with flat apical edging; veins and claval suture absent; pronotum about 2.5–2.8 × as long as wing commissure; metathoracic spiracle surrounded by distinct microsculpture; scent gland evaporatory area roughly triangular, with flat peritreme (Figure 17); hind femur enlarged and somewhat flattened, distinctly surpassing apex of abdomen, pretarsus as in Figs 22, 23, with smoothly curved claws and fleshy, apically convergent parempodia, pulvilli absent. Abdomen constricted at base in lateral view and greatly expanded posterior to basal segments. *Genitalia*: genital capsule wide, heavily sclerotized, partly retracted into pregenital segments, without distinctive ornamentation or processes (Figure 24), ventral wall with narrow cone-shaped extension caudally, genital opening wide, oval; parameres of typical halticine shape, left paramere sickle-shaped, gradually curving and terminating with oblique T-shaped (Figs 30, 34) or harpoon-shaped (Figs 41, 44) blade; right paramere larger than left one, with long basal process, apically flattened and concave, flag-shaped, subquadrate in lateral view, with apical denticle on inner margin (Figs 33, 36, 43, 46); phallotheca of aedeagus (Figs 38, 40, 48, 50) voluminous, with dorsal wall entirely sclerotized, upturned apically, ventral wall sclerotized apically, membranous at base, equipped with pair of sclerotized and rounded subapical outgrowths at sides; ductus seminis relatively short, basal half membranous, coiled, with distinct sclerotized rings, apical half somewhat wider, straight and heavily sclerotized, terminating in horseshoe-shaped, sculptured secondary gonopore; endosoma without sclerites, membranous, folded, with several eversible, finely dentate and sometimes slightly sclerotized lobes (Figs 37, 39, 47, 49).

***Female***. Similar to male in coloration, surface, and main structural details, but differing in structure and vestiture of antennal segments, abdomen strongly expanded at middle, and body proportions (Figs 8–10, 12, 13). Body larger on average, total length 2.9–4.5. *Coloration*: As in male but antennal segment I usually dirty yellow, rarely dark brown, segments III and IV black, sometimes partly or entirely dirty pale brown; femora entirely dirty yellow to dark brown with paler apices. *Surface and vestiture*: As in male, but frons and clypeus without long, thin, whitish setae and black spinelike setae; antennal segment I with regularly distributed, minute, black, adpressed simple setae and 6–12 spinelike setae on mesial surface; segments II-IV with mixture of relatively scarce erect black setae and dense, semierect, pale and thin simple setae. *Structure*: Similar to male, but antenna not modified, segment I short, slightly and uniformly swollen, at most twice as wide as second, segment II rod-shaped, straight; labium always reaching and usually surpassing hind coxa; abdomen more expanded at middle than in male, broadly oval in lateral and dorsal view. *Genitalia*: dorsal labiate plate of bursa copulatrix membranous, with inconspicuous anterior and posterior margins and medially recurved lateral margins, without additional sclerotization and microtrichia; sclerotized ring broadly oval to subtriangular, more or less strongly curved along longitudinal axis, sometimes with strongly and narrowly elongate apex (Figs 51–54); posterior wall membranous, with almost flat, minutely dentate or coarsely rugose interramal sclerites (Figs 55–58); inner margins of first gonapophyses vestibulum with simple and symmetrical, roughly triangular sclerites encircling vulva (Figure 63); first gonapophysis distinctly sagittate, second gonapophysis saber-shaped, gradually tapering.

##### Distribution.

The subgenus is endemic to the Caucasus Mountains including Greater and Lesser Caucasus, and Armenian Highlands (Figure 64). Given the recent records of Caucasian halticines in the Pontic Mountains (Dursun and Kartal 2006), species of *Plumiger* eventually might be found there.

##### Discussion.

The striking sexual dimorphism in *Plumiger* species and one of the main diagnostic features of the subgenus, is exhibited by the enlarged antennal segments I and II and their peculiar vestiture (Figs 18, 19). Males apparently use their antennae to grasp females during copulation in a manner similar to that of several other plant bug genera with sexually dimorphic antennae, e.g., *Harpocera* Curtis, 1838 (Kullenberg 1944, Stork 1981) and *Spanagonicus* Berg, 1883. The second antennal segment II of the latter genus is also equipped with spatulate setae that may have an adhesive function (Menard 2015).

##### Natural history.

We swept specimens of *Plumiger* in large numbers between 1950 and 2450 m a.s.l. in different grasslands and steppe habitats, viz. Ponto–Caucasian hay meadows, grass meadow-steppes, feather-grass steppes, and acid subalpine grasslands (Figs 1–4). *Myrmecophyesarmeniacus* and *M.heterocerus* studied were associated with Poaceae. Occasionally adults were found on species of Asteraceae. Although no host records are available for *M.nasutus* and *M.tomi* sp. n., the label data indicate that they are confined to highland grasslands. Nearly all species of *Myrmecophyes* s. str. with known host associations also utilize Poaceae, typically occur in large numbers and are considered pests of pastures across highlands of Central Asia (Bykov 1971). In contrast, *M.trispiculus* and *M.geniculatus* feed on *Artemisia* spp. (Drapolyuk and Kerzhner 2000).

Almost nothing is known about the phenology of *Plumiger* species. Based on the presence of eggs in females, the ratio of males to females, and available collection dates, we speculate that *M.armeniacus* and *M.heterocerus* have one and two generations, respectively. More interesting are our observations of diurnal activity of Armenian *Myrmecophyes*. Despite maximal efforts in suitable habitats, we had little success in collecting at noon. The insects became active and started to move up the culm and leaves of grasses approximately two and a half hours before sunset (just after 18:00 in June). During the day, the bugs were close to the surface, hidden among lower parts of the grasses. This corresponds with our previous observations on other brachypterous halticines, viz. *Myrmecophyes* s. str., *Scirtetellus*, and *Dimorphocoris* spp. in the high mountains of the Balkans and Central Asia. Schuh and Lattin (1980) reported plausible midmorning and late-afternoon peaks of activity for *M.oregonensis* sampled southwest of Burns, Oregon.

### Key to males

**Table:** 

1	Clypeus distinctly bulging, rectangular in lateral view (Figs 27, 29). Antennal segment II longer than pronotal width at middle and 1.8–1.9 × as long as segment I. Left paramere harpoon-shaped, with one or two recurved denticles apically (Figs 41, 44). Endosoma of aedeagus with three folded and slightly sclerotized lobes (Figs 47, 49)	**3**
-	Clypeus not bulging, slightly convex in apical half (Figs 26, 28). Antennal segment II shorter than width of pronotum at middle and 1.2–1.4 × as long as segment I. Left paramere ending with oblique T-shaped blade (Figs 30, 34). Endosoma of aedeagus with entirely membranous lobes (Figs 37, 39)	**2**
2	Larger, body length 3.4–3.7. Head and abdomen matt. Left paramere with rounded outgrowth at base of apical process (Figs 30, 31)	*** armeniacus ***
-	Smaller, body length 2.8–3.2. Head and abdomen smooth and shiny. Left paramere without outgrowth at base of apical process (Figure 35)	*** heterocerus ***
3	Antennal segments longer, segment I 1.3–1.4 × as long as pronotum, segment II 1.2–1.3 × as long as head width and 1.8–1.9 × as long as width of pronotum at middle. Left paramere with two subapical recurved denticles (Figs 41, 42)	*** nasutus ***
-	Antennal segments shorter, segment I 0.9 × as long as pronotum, segment II 0.9 × as long as head width and 1.2 × as long as width of pronotum at middle. Left paramere with single subapical recurved denticle (Figs 44, 45).	*** tomi ***

### Key to females

**Table:** 

1	Larger, body length 3.9–4.5. Head and abdomen matt (Figs 8, 12, 13)	**2**
-	Smaller, body length 2.8–3.2. Head and abdomen smooth and shiny (Figs 9, 10).	**3**
2	Body length 3.9–4.1. Sclerotized rings of dorsal labiate plate broadly oval (Figs 51, 59)	*** armeniacus ***
-	Body length 4.2–4.5. Sclerotized rings of dorsal labiate plate subtriangular, long and narrow, with apices distinctly attenuated (Figs 54, 61)	*** nasutus ***
3	Sclerotized rings of dorsal labiate plate subtriangular, long and narrow, with apices distinctly attenuated (Figs 53, 60)	*** heterocerus ***
-	Sclerotized rings of dorsal labiate plate broadly oval (Figs 52, 62)	*** tomi ***

#### Myrmecophyes armeniacus

Taxon classificationAnimaliaHemipteraMiridae

Drapolyuk, 1989

Figs 5 , 8 , 14 , 16 , 22 , 23 , 25 , 26 , 30–33 , 37 , 38 , 51 , 55 , 59 , 64

Myrmecophyes (Plumiger) armeniacus Dapolyuk, 1989: 125; figs 51, 55.

##### Material examined.

**Holotype**: **TURKEY: Kars**: Kars, 40.58333°N, 43.06666°E, 04 Jun 1915, Olsufiev, 1♀ (AMNH_PBI 00261681) (ZISP). **Paratypes**: **ARMENIA: Shirak**: Gyumri [Leninakan], 40.78333°N, 43.83333°E, 16 Jul 1940, Esterberg, 1♀ (AMNH_PBI 00261680) (ZISP).

##### Other specimens examined:

**ARMENIA: Gegharkunik**: N coast of Sevan Lake, Artanish – Shorzha Rd, 40.50109°N, 45.3282°E, 1962 m, 14 Jun 2017, F. Konstantinov & N. Simov, (Poaceae), 15♂ (AMNH_PBI 00343264-AMNH_PBI 00343277, AMNH_PBI 00343331), 70♀ (AMNH_PBI 00343249-AMNH_PBI 00343263, AMNH_PBI 00343232-AMNH_PBI 00343248, AMNH_PBI 00343196-AMNH_PBI 00343231, AMNH_PBI 00343330, AMNH_PBI 00343376) (ZISP); 16♂, 49♀ (SOFM). N coast of Sevan Lake, Karmir Pass, 4 km ENE of Aghberk, 40.56328°N, 45.29903°E, 2190 m, 15 Jun 2017, F. Konstantinov & N. Simov, (Poaceae), 2♂ (AMNH_PBI 00343351, AMNH_PBI 00343352), 1♀ (AMNH_PBI 00343327) (ZISP); 6♂, 9♀ (SOFM). W coast of Sevan Lake, 2 km ESE of Semyonovka, 40.65074°N, 44.92336°E, 2123 m, 15 Jun 2017, F. Konstantinov & N. Simov, (Poaceae), 24♂ (AMNH_PBI 00343278-AMNH_PBI 00343285, AMNH_PBI 00343288-AMNH_PBI 00343301, AMNH_PBI 00343332, AMNH_PBI 00343334), 25♀ (AMNH_PBI 00343302-AMNH_PBI 00343312, AMNH_PBI 00343314-AMNH_PBI 00343325, AMNH_PBI 00343333, AMNH_PBI 00343328) (ZISP); 25♂, 34♀ (SOFM).

##### Diagnosis.

Size large, clypeus straight, not bulging apically in either sex (Figs 14, 26), head and abdomen not shiny (Figs 5, 8), antennal segment II in male shorter than width of pronotum; shape of the left paramere as in Figs 30–32, lobes of aedeagus membranous (Figure 37), and sclerotized rings of dorsal labiate plate broadly oval (Figs 51, 55).

##### Remarks.

Males of *M.armeniacus* are most similar to *M.heterocerus* in structure of the head and male genitalia, but the latter species can be distinguished by the smaller size, shiny head and abdomen, and structure of the left paramere with the apical process narrowing before the T-shaped blade and equipped with a prominent outgrowth at base (compare Figs 30, 34). Females of *M.armeniacus* and *M.nasutus* are roughly similar in size and vestiture, and the surface of the dorsum is not shiny; these species can be separated by their differently shaped sclerotized rings (compare Figs 51, 54).

##### Redescription.

***Male***. Total length 3.4–3.7. *Coloration*: (Figure 5): entirely black, with whitish transverse stripe along apex of wing pad; fore and middle femora usually, hind femur rarely with pale brown apices. *Surface and vestiture*: Dorsum matt, head and abdomen at most moderately shiny; vestiture as in subgeneric description. *Structure*: Body 3.9–4.2 × as long as width of pronotum at middle. Head: Clypeus not bulging (Figure 26), flat at base and slightly convex in apical part, with apical margin straight; vertex 1.8–2.0 × as wide as eye; antennal segment I strongly swollen, short, 0.8–0.9 × as long as length of pronotum, segment II slightly curved and widened apically, rather short, 1.4–1.5 × as long as I, 0.8 × as long as width of pronotum at middle, and 0.6 × as long as width of head; segment III more than 2.8–2.9 × as long as II and distinctly longer than I and II combined; segment IV about 1.5 × as long as II. Thorax: Pronotum at middle 1.3 × as wide as long, 0.7–0.8 × as wide as head; hind tibia 3.6–4.0 × as long as width of pronotum at middle. *Genitalia*: genital capsule comparatively small, about 0.3 of abdomen; left paramere sickle-shaped, thin, apical process of uniform width along entire length, long, somewhat sinuate, flattened, ending with characteristic oblique T-shaped blade; body of paramere with small but distinct rounded outgrowth at base of apical process with long setae (Figs 30–32); right paramere typically flag-shaped, somewhat larger than in *M.heterocerus* (Figure 33); endosoma of aedeagus voluminous, folded, entirely membranous, with several small, minutely dentate eversible lobes (Figs 37, 38).

***Female***. Body large, total length 3.9–4.1. *Coloration*: As in male, but antennal segment I and femora ranging from uniformly rust brown to totally black (Figure 8). *Surface and vestiture*: As in subgeneric description; dorsum matt, head and abdomen at most moderately shiny; frons and vertex with few erect spinelike setae; antennal segment I with 6–8 similar setae on mesial surface. *Structure*: Body 4.4–4.6 × as long as width of pronotum at middle. Head: Vertex 1.9–2.1 × as wide as eye; antennal segment I 0.8 × as long as length of pronotum, segment II 1.9–2.1 × as long as I, 1.1–1.2 × as long as width of pronotum at middle, and 0.8–0.9 × as long as width of head; segment III 1.5–1.6 × as long as II; segment IV equal to II in length. Thorax: Pronotum at middle 1.3–1.4 × as wide as long, 0.7–0.8 × as wide as head; hind tibia 3.3–3.5 × as long as width of pronotum at middle. *Genitalia*: Sclerotized rings of dorsal labiate plate broadly oval (Figs 51, 59), interramal sclerites of posterior wall almost flat, densely covered with minute denticles (Figure 55).

##### Distribution.

The current distribution of *M.armeniacus* spans a distance of less than 200 km from northeastern Turkey in the west to northwestern Armenia in the east (Figure 64).

#### Myrmecophyes heterocerus

Taxon classificationAnimaliaHemipteraMiridae

Horváth, 1927

Figs 6 , 9 , 15 , 17–19 , 20–21 , 24 , 28 , 34–36 , 39 , 40 , 53 , 56 , 60 , 63 , 64

Myrmecophyes heterocerus Horváth, 1927: 189
Myrmecophyes (Plumiger) heterocerus Drapolyuk, 1989: 135, figs 46–50, 54 (figs, habitus, male and female genitalia)

##### Material examined.

**ARMENIA: Gegharkunik**: 7 km S from Nshkhark, 39.95378°N, 45.23438°E, 2418 m, 13 Jun 2017, F. Konstantinov & N. Simov, (Poaceae), 56♀ (AMNH_PBI 00343163-AMNH_PBI 00343195, AMNH_PBI 00343338, AMNH_PBI 00343339, AMNH_PBI 00343156-AMNH_PBI 00343162, AMNH_PBI 00343149-AMNH_PBI 00343155, AMNH_PBI 00343142-AMNH_PBI 00343148), 70♂ (AMNH_PBI 00343378, AMNH_PBI 00343337, AMNH_PBI 00343329, AMNH_PBI 00343071-AMNH_PBI 00343074, AMNH_PBI 00343069, AMNH_PBI 00343067, AMNH_PBI 00343080-AMNH_PBI 00343136, AMNH_PBI 00343138-AMNH_PBI 00343141), 2 larvae (AMNH_PBI 00343075, AMNH_PBI 00343137) (ZISP); 45♂, 49♀ (SOFM). **AZERBAIJAN: Kalbajar**: Istisu nr Kalbacar [Kel’badzhary], 39.96972°N, 45.95°E, 03 Jul 1968 - 04 Jul 1968, Gidayatov, 3♂ (AMNH_PBI 00271255, AMNH_PBI 00261439, AMNH_PBI 00261440), 4♀ (AMNH_PBI 00261638, AMNH_PBI 00261637) (ZISP). **GEORGIA: Mtskheta-Mtianeti**: Kobi, Voenno-Gruzinskaya doroga [=Georgean Military Rd], 42.5583°N, 44.5122°E, 2144 m, 14 Jul 1925, Kiritshenko, 3♂ (AMNH_PBI 00261433-AMNH_PBI 00261435), 14♀ (AMNH_PBI 00261608-AMNH_PBI 00261621) (ZISP); 15 Jul 1925, A. N. Kiritshenko, 14♀ (AMNH_PBI 00271256-AMNH_PBI 00271259, AMNH_PBI 00261622-AMNH_PBI 00261631), 1♂ (AMNH_PBI 00261436) (ZISP). **RUSSIAN FEDERATION: North Ossetia Rep.**: Pass between Verkhnyaya Kora and Ardon Rivers, 42.82306°N, 43.91583°E, 01 Aug 1925, A. N. Kiritshenko, 6♀ (AMNH_PBI 00271254, AMNH_PBI 00261633-AMNH_PBI 00261636, AMNH_PBI 00261438), 1♂ (AMNH_PBI 00261437) (ZISP). Verkhniy Tsey, 42.80667°N, 43.94028°E, 04 Aug 1925, A. N. Kiritshenko, 1♀ (AMNH_PBI 00261632) (ZISP).

##### Diagnosis.

Distinguished from *M.armeniacus* by the smaller size, shiny abdomen (Figs 6, 9), shape of left paramere (Figs 34, 35) and narrowly triangular sclerotized rings of dorsal labiate plate (Figs 53, 60). The diagnosis of *M.armeniacus* provides additional characters.

##### Redescription.

***Male***. Total length 2.8–3.2. *Coloration*: As in *M.armeniacus*. *Surface and vestiture*: Pronotum and scutellum matt, head and abdomen smooth, distinctly shiny (Figure 6); vestiture as in subgeneric description. *Structure*: Body 3.9–4.5 × as long as width of pronotum at middle. Head: Clypeus not bulging (Figure 28), slightly convex in apical half, with shallow depression at base and apical margin straight; vertex 1.6–1.9 × as wide as eye; antennal segment I strongly swollen, short, 0.8–0.9 × as long as length of pronotum, segment II slightly curved and widened apically, short, 1.2–1.3 × as long as I, 0.7–0.8 × as long as width of pronotum at middle, and 0.5–0.6 × as long as width of head; segment III more than 2.5 × as long as II and distinctly longer than I and II combined; segment IV about 1.5 × as long as II. Thorax: Pronotum at middle 1.3–1.4 × as wide as long, 0.7 × as wide as head; hind tibia 3.0–3.5 × as long as width of pronotum at middle. *Genitalia*: genital capsule comparatively small, about 0.3 of abdomen; left paramere sickle-shaped, thin, apical process straight, narrowed subapically, ending with characteristic oblique T-shaped blade; body of paramere without outgrowth at base of apical process (Figs 34, 35); right paramere typically flag-shaped, somewhat smaller than in *M.armeniacus* (Figure 36); endosoma of aedeagus voluminous, folded, entirely membranous, with several small, minutely dentate eversible lobes (Figs 39, 40).

***Female***. Similar to male but body larger on average, total length 2.9–3.8. *Coloration*: As in male, but with pale brown to dirty yellow antennal segment I (Figure 9). *Surface and vestiture*: As in subgeneric description; pronotum and scutellum matt, head and abdomen distinctly shiny; frons and vertex with few erect spinelike setae; antennal segment I with 6–8 similar setae on mesial surface. *Structure*: Body 3.6–4.3 × as long as width of pronotum at middle. Head: Vertex 1.9–2.1 × as wide as eye; antennal segment I 0.8–0.9 × as long as length of pronotum, segment II 1.7–2.0 × as long as I, 1.0–1.1 × as long as width of pronotum at middle, and 0.8–0.9 × as long as width of head; segment III 1.3–1.4 × as long as segment II; segment IV equal to II in length. Thorax: Pronotum at middle 1.4–1.5 × as wide as long, 0.7–0.8 × as wide as head; hind tibia 2.8–3.0 × as long as width of pronotum at middle. *Genitalia*: Sclerotized rings of dorsal labiate plate narrow, subtriangular, with apices narrowly elongate (Figs 53, 60); interramal sclerites flat, finely dentate at base (Figure 56).

##### Distribution.

*Myrmecophyesheterocerus* appears to be the most wide-ranging species of the subgenus, occurring along the Greater Caucasus range from North Ossetia in the west across Georgia to southeastern Dagestan in the east, and along the Greater Caucasus range to central Armenia and western Azerbaijan in the south (Figure 64).

#### Myrmecophyes nasutus

Taxon classificationAnimaliaHemipteraMiridae

Drapolyuk, 1989

Figs 11–13 , 27 , 41–43 , 47 , 48 , 54 , 57 , 61 , 64

Myrmecophyes (Plumiger) nasutus Drapolyuk, 1989: 125; figs 40–45, 52, 53.

##### Material examined.

**Holotype**: **GEORGIA**: **Racha-Lechkhumi and Kvemo Svaneti**: Korel’dash, 42.9183°N, 43.144°E, 26 Jul 1957, Akramovskaya, 1♂ (AMNH_PBI 00261441) (ZISP). **Paratypes**: **GEORGIA: Racha-Lechkhumi and Kvemo Svaneti**: Korel’dash, 42.9183°N, 43.144°E, 26 Jul 1957, Akramovskaya, 2♀ (AMNH_PBI 00271200, AMNH_PBI 00271201) (ZISP); 30 Jul 1957, Akramovskaya, 1♂ (AMNH_PBI 00261442), 23♀ (AMNH_PBI 00261639-AMNH_PBI 00261661) (ZISP). Lentekhi, Svanetia, 42.78333°N, 42.7°E, 10 Aug 1957, Akramovskaya, 3♀ (AMNH_PBI 00261674-AMNH_PBI 00261676) (ZISP). Tsena, Svanetia, 42.8789°N, 43.1519°E, 25 Jul 1957, Akramovskaya, 15♀ (AMNH_PBI 00271197-AMNH_PBI 00271199, AMNH_PBI 00261662-AMNH_PBI 00261673) (ZISP). Zarsky Pass, Lentekhi Distr., 42.91666°N, 43.16666°E, 27 Jul 1957, Akramovskaya, 3♀ (AMNH_PBI 00261677-AMNH_PBI 00261679) (ZISP). **Mtskheta-Mtianeti**: Kazbek Mt., 42.68333°N, 44.46666°E, 17 Jul 1910, Unknown collector, 1♀ (AMNH_PBI 00261685) (ZISP).

##### Diagnosis.

Distinguished from other *Plumiger* spp. by the larger size (Figs 11–13), distinctly bulging clypeus (Figure 27), non-shiny head and abdomen, harpoon-shaped left paramere with two subapical recurved denticles (Figs 41, 42), and long, narrow, distinctly attenuate sclerotized rings of dorsal labiate plate (Figs 54, 57). The diagnosis and remarks for *M.tomi* include a discussion of the distinctive features.

##### Redescription.

***Male***. Total length 3.8–3.9. *Coloration* (Figure 11): Black, with pale brown apices of femora and dirty white transverse stripe along apex of wing pad. *Surface and vestiture*: As in generic diagnosis. *Structure*: Body 4.2–4.6 × as long as width of pronotum at middle. Head: Clypeus strongly bulging apically, concave and apically rectangular in lateral view (Figure 27), apically dilated, broadly rounded in frontal view; vertex 2.1–2.2 × as wide as eye; antennal segment I strongly swollen, relatively long, 1.3–1.4 × as long as length of pronotum, segment II gradually curved along entire length and somewhat flattened apically, long, 1.8–1.9 × as long as I, 1.8–1.9 × as long as width of pronotum at middle, and 1.2–1.3 × as long as width of head. Thorax: Pronotum at middle 1.3–1.5 × as wide as long, 0.7 × as wide as head; hind tibia 3.8–4.2 × as long as width of pronotum at middle. *Genitalia*: genital capsule about 0.4 of abdomen; left paramere as in *M.tomi* but slightly larger, with two subapical recurved denticles (Figs 41, 42); right paramere similar to that of *M.tomi* but larger and equipped with larger apical tooth on inner margin (Figure 43); endosoma of aedeagus voluminous, membranous, with three large, folded, minutely dentate and slightly sclerotized eversible lobes (Figs 47, 48).

***Female***. Body larger on average, total length 4.2–4.5. *Coloration*: Similar to male, but antennal segment I ranging from dirty orange to entirely black, coxae usually entirely or at least apically dirty orange, femora from entirely dirty orange to black, with apices dirty orange (Figs 12, 13). *Surface and vestiture*: As in *M.tomi* sp. n. Structure: Body 4.1–4.6 × as long as width of pronotum at middle. Head: Clypeus convex, not bulging apically; vertex 2.0–2.4 × as wide as eye; antennal segment I slightly and uniformly swollen, short, equal to length of pronotum, segment II thin, straight and rod-shaped, 2.2–2.4 × as long as I, 1.4–1.6 × as long as width of pronotum at middle, and 1.1–1.2 × as long as width of head. Thorax: Pronotum at middle 1.5 × as wide as long, 0.7-.0.8 × as wide as head; hind tibia 3.2–3.3 × as long as width of pronotum at middle. *Genitalia*: Sclerotized rings of dorsal labiate plate long and narrow, gradually curved, with apices gradually attenuated (Figs 54, 61); interramal sclerites flat, finely striated (Figure 57).

##### Distribution.

Known from several localities in northern Georgia along the southern slopes of the Greater Caucasus Mountain range (Figure 64).

##### Discussion.

Drapolyuk (1989) diagnosed *M.nasutus* as having the aedeagus with a single spicula, although her figure 45 shows a membranous and finely dentate lobe of the endosoma. We observed that the eversible lobes of the endosoma in *M.nasutus* and *M.tomi* sp. n. are faintly sclerotized but effectively devoid of spiculae.

#### Myrmecophyes tomi sp. n.

Taxon classificationAnimaliaHemipteraMiridae

http://zoobank.org/A15DCD84-E454-4AE3-8B26-8021EF144F14

Figs 7 , 10 , 29 , 44–46 , 49 , 50 , 52 , 58 , 62

##### Type locality.

Georgia, Kakheti, Sakhkhova Mts. Range, 42.36666°N, 45.61666°E

##### Type material.

**Holotype**: **GEORGIA: Kakheti**: Sakhkhova Mts. Range, Tushetia, 42.36666°N, 45.61666°E, 28 Aug 1959, I. Zaytseva, 1♂ (AMNH_PBI 00261443) (ZISP). **Paratypes**: **GEORGIA: Kakheti**: Sakhkheva Mts. Range, Tushetia, 42.36666°N, 45.61666°E, 28 Aug 1959, I. Zaytseva, 1♀ (AMNH_PBI 00261682) (ZISP). **Shida Kartli (South Ossetia)**: Tskaro, 22 Jun, Demokidov, 1♀ (AMNH_PBI 00261684) (ZISP). **RUSSIAN FEDERATION: Dagestan Rep.**: Khochaldag Mt., 42.73333°N, 46.26666°E, 18 Jul 1909, A. Mlokossiewich, 1♂ (AMNH_PBI 00261444), 3♀ (AMNH_PBI 00261686-AMNH_PBI 00261688) (ZISP); 12 Aug 1910, A. Mlokossiewich, 1♀ (AMNH_PBI 00261683) (ZISP).

##### Diagnosis.

Distinguished from congeners by the small size (Figs 7, 10), distinctly bulging clypeus in male (Figure 29), antennal segments I and II in male not exceeding length and width of pronotum respectively, shiny head and abdomen, characteristically harpoon-shaped left paramere (Figs 44, 45), faintly sclerotized lobes of endosoma (Figure 49), and broadly oval, weakly attenuate sclerotized rings (Figs 52, 58).

##### Remarks.

*Myrmecophyestomi* is separated from the morphologically similar *M.nasutus* by the shiny head and abdomen, the smaller body size, the shorter first two antennal segments in the male, the single subapical, recurved denticle of the left paramere, and the broad sclerotized rings. The last-named character would allow females of *M.tomi* to be distinguished from those of *M.heterocerus*, which otherwise are similar in body size and proportions, and in having a shiny abdomen.

**Description**. ***Male***. Total body length 3.4. *Coloration*: Dorsum and venter uniformly black, with contrasting yellowish-white stripe along apical margin of wing pad; antennal segment I chestnut brown, remaining segments dark brown; coxae and femora dirty yellow, tibiae and tarsi dark brown (Figure 7). *Surface and vestiture*: Head, pronotum and hemelytron rugose, pronotum with fine transverse wrinkles along anterior and posterior margin; abdomen shiny, distinctly smoother than pronotum and scutellum. Dorsum, venter, and legs with very short and thin, reclining, pale brown simple setae, scarce on head, thorax and femora, more dense on abdomen and tibiae; head with several spinelike setae on vertex posteriorly and on frons between eye and antennal fossa, apex of frons and base of clypeus with very dense, thin and exceptionally long, straight whitish setae; antennal segments I and II with numerous black spinelike setae on dorsal surfaces and dense, white, spatulate scales on ventral surfaces except basal one-fourth; segments III and IV with relatively scarce, erect, spinelike setae shorter than those on previous segments and dense, semierect, pale simple setae; femora with incomplete row of stiff spinelike setae along dorsal margin and several apical spines dorsally and ventrally; tibia with scattered black spines. *Structure*: Body 4.0 × as long as width of pronotum at middle. Head: Vertical, with slightly concave and apically bulging clypeus, rectangular in lateral view (Figure 29) and with somewhat dilated, broadly rounded apex in frontal view; vertex 2.0 × as wide as eye; antennal segment I distinctly swollen, 0.9 × as long as length of pronotum, segment II gradually curved along entire length, somewhat widened and flattened apically, relatively long, 1.8 × as long as I, 1.2 × as long as width of pronotum at middle, and 0.9 × as long as width of head; remaining segments thin, segment III twice as long as segments II and IV; labium stout, surpassing hind coxa and reaching basal abdominal segments. Thorax: Pronotum at middle 1.3 × as wide as long, 0.8 × as wide as head, metathoracic spiracle and scent efferent system as in *M.heterocerus* (Figure 17); hind tibia 3.8 × as long as width of pronotum at middle; tarsal segments II and III subequal in length, about 1.5 × as long as segment I, pretarsus as in *M.armeniacus* (Figs 22, 23). *Genitalia*: genital capsule about 0.4 of abdomen; left paramere sickle-shaped, comparatively robust, apical process harpoon-shaped, slightly curved, roughly triangular in cross section, tapering towards apex, with subapical recurved denticle; body of paramere with distinct rounded outgrowth at base of apical process with long setae (Figs 44, 45); right paramere typically flag-shaped, with minute apical tooth on inner margin (Figure 46); endosoma of aedeagus membranous, similar to that of *M.nasutus* but somewhat smaller, with three folded, minutely dentate and slightly sclerotized eversible lobes (Figs 49, 50).

***Female***. Larger than male on average, total body length 3.1–3.5, with antenna unmodified and abdomen broadly oval, strongly expanded at middle. *Coloration*: Similar to male, but antennal segment I dirty orange, coxae and femora black, with apices dirty orange (Figure 10). *Surface and vestiture*: Similar to male, but frons without dark erect spinelike setae and long whitish thin setae; antennal segments I and II without scales and long spinelike setae, segment I with only 8–12 relatively small spinelike erect setae on mesial surface. *Structure*: Body 3.8–4.5 × as long as width of pronotum at middle. Head: Clypeus convex, not bulging apically; vertex 1.9–2.1 × as wide as eye; antennal segment I slightly and uniformly swollen, short, 0.7–0.9 × as long as length of pronotum, segment II straight and thin, rod-shaped, 1.7–2.0 × as long as I, 1.0–1.1 × as long as width of pronotum at middle, and 0.7–0.8 × as long as width of head; remaining segments filiform, segment III about 1.5 × as long as segments II and IV. Thorax: Pronotum at middle 1.4–1.5 × as wide as long, 0.7–0.8 × as wide as head; labium always reaching and usually surpassing hind coxa; hind tibia 2.8–3.3 × as long as width of pronotum at middle. *Genitalia*: Sclerotized rings of dorsal labiate plate elongate-oval (Figs 52, 62); interramal sclerites convex and covered with dense minute denticles (Figure 58).

##### Etymology.

The species is named for Thomas J. Henry in recognition of his unparalleled contributions to heteropterology.

##### Distribution.

Known from northeastern Georgia in the Transcaucasia to southwestern Dagestan in the North Caucasus (Figure 64).

##### Discussion.

The new species is described from the specimens originally included in the paratype series of *M.nasutus* by Drapolyuk (1989), who discussed distinctions in the structure of antennal segment I in male and figured the dorsal labiate plate (Drapolyuk 1989: fig. 53), but refrained from describing a new taxon.

**Figures 14–19.**
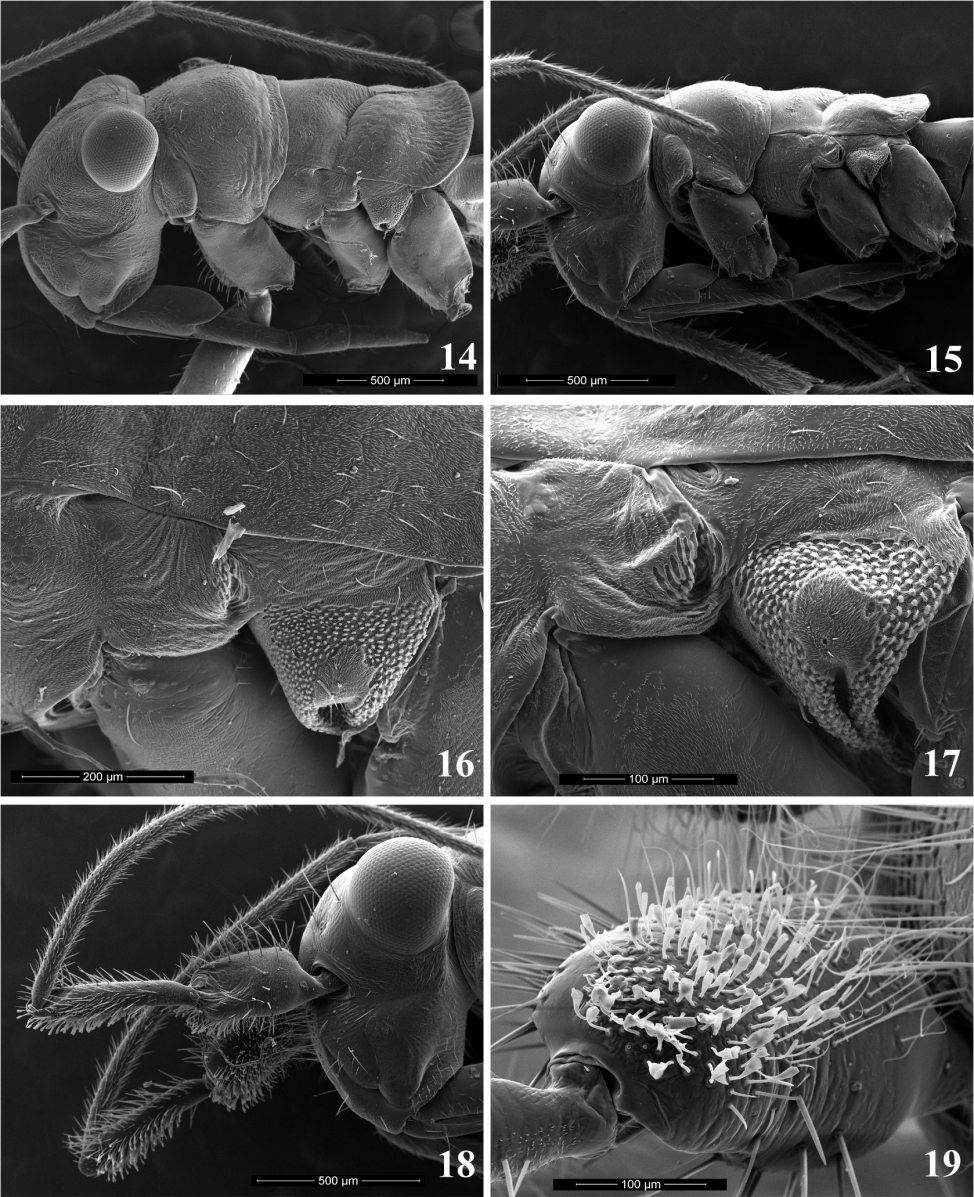
Scanning electron micrographs. **14, 15** Head and thorax in lateral view **14***Myrmecophyesarmeniacus* Drapolyuk, 1989, female **15***Myrmecophyesheterocerus* Horváth, 1927, male **16, 17** Metathoracic scent gland evaporative area and spiracle **16***M.armeniacus***17***M.heterocerus***18** Head of *M.heterocerus* male in lateral view **19** Antennal segment I of *M.heterocerus* male.

**Figures 20–25.**
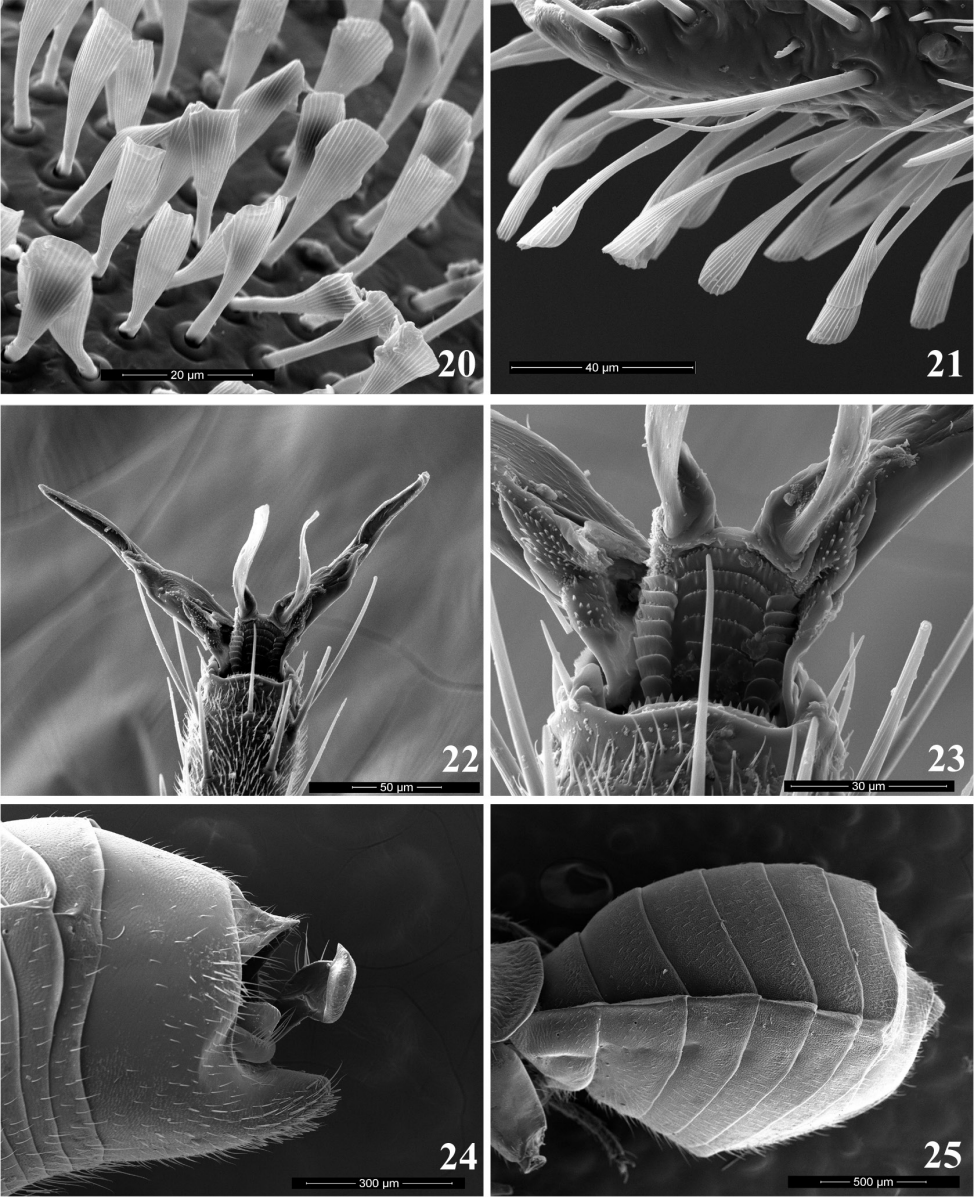
Scanning electron micrographs. **20, 21** Vestiture of male antennal segments in *Myrmecophyesheterocerus* Horváth, 1927 **20** segment I **21** segment II **22, 23** Pretarsus of *Myrmecophyesarmeniacus* Drapolyuk, 1989 **24** Genital segment of *M.heterocerus* male **25** Abdomen of *M.armeniacus* female.

**Figures 26–29.**
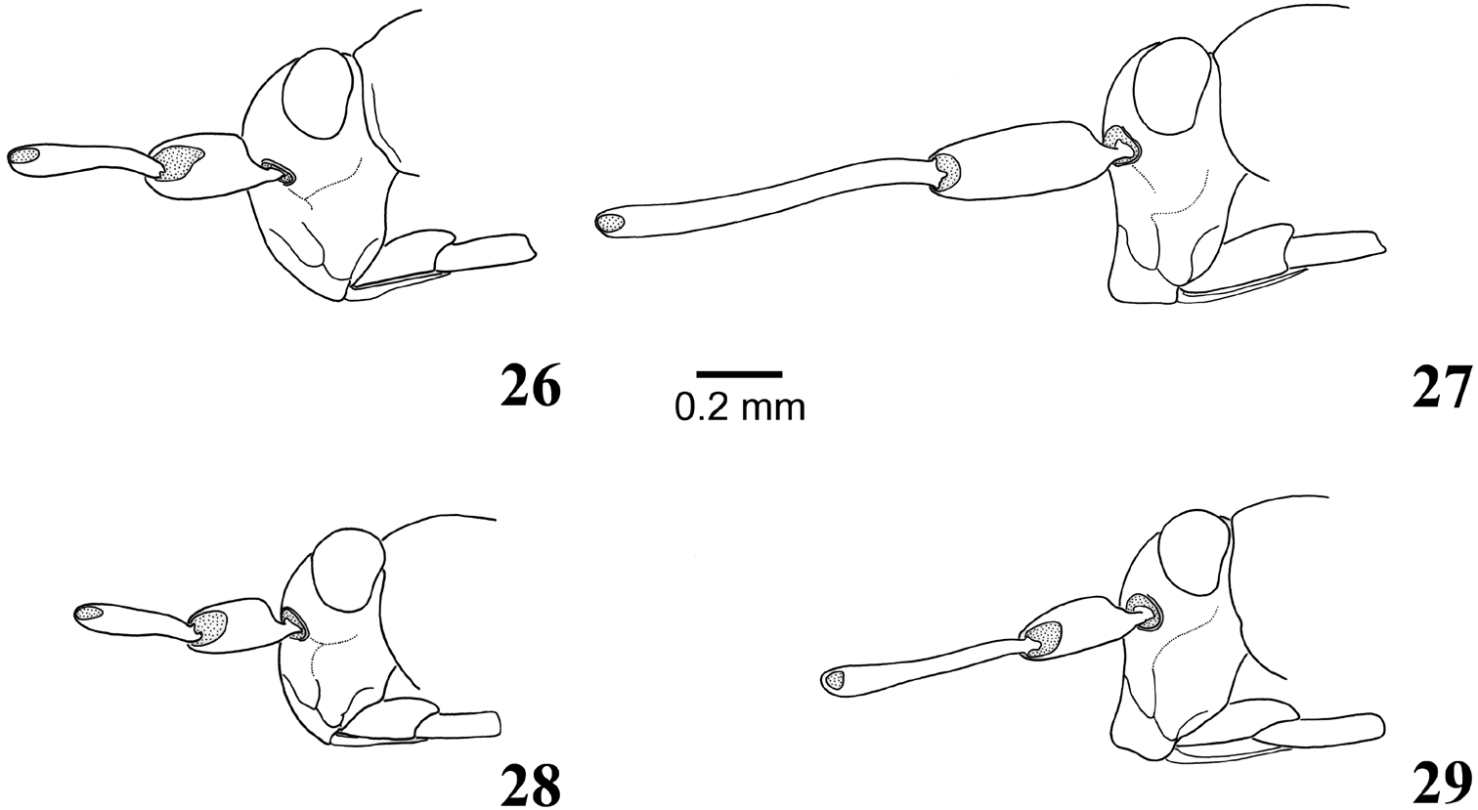
Male head in lateral view: **26***Myrmecophyesarmeniacus* Drapolyuk, 1989 **27***Myrmecophyesnasutus* Drapolyuk, 1989 **28***Myrmecophyesheterocerus* Horváth, 1927 **29***Myrmecophyestomi* sp. n.

**Figures 30–40.**
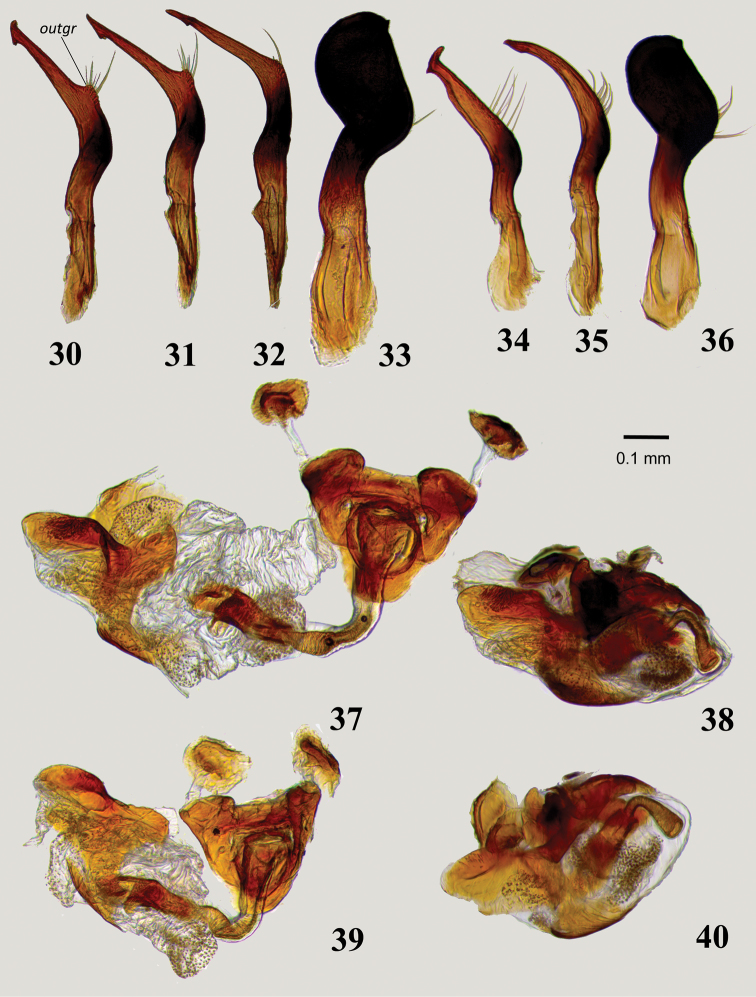
Male genitalia: **30–32, 34–35** Left paramere in lateral (**30, 34**) and dorsal (**31, 32, 35**) views **30–32***Myrmecophyesarmeniacus* Drapolyuk, 1989 **34, 35***Myrmecophyesheterocerus* Horváth, 1927 **33, 36** Right paramere in dorsal view **33***M.armeniacus***36***M.heterocerus***37–40** Aedeagus in lateral view (**38, 40**) and with phallotheca detached from phallobase and partially expanded endosoma (**37, 39**) **37, 38***M.armeniacus***39, 40***M.heterocerus*. Abbreviations: *outgr* – rounded outgrowth at base of apical process of the left paramere.

**Figures 41–50.**
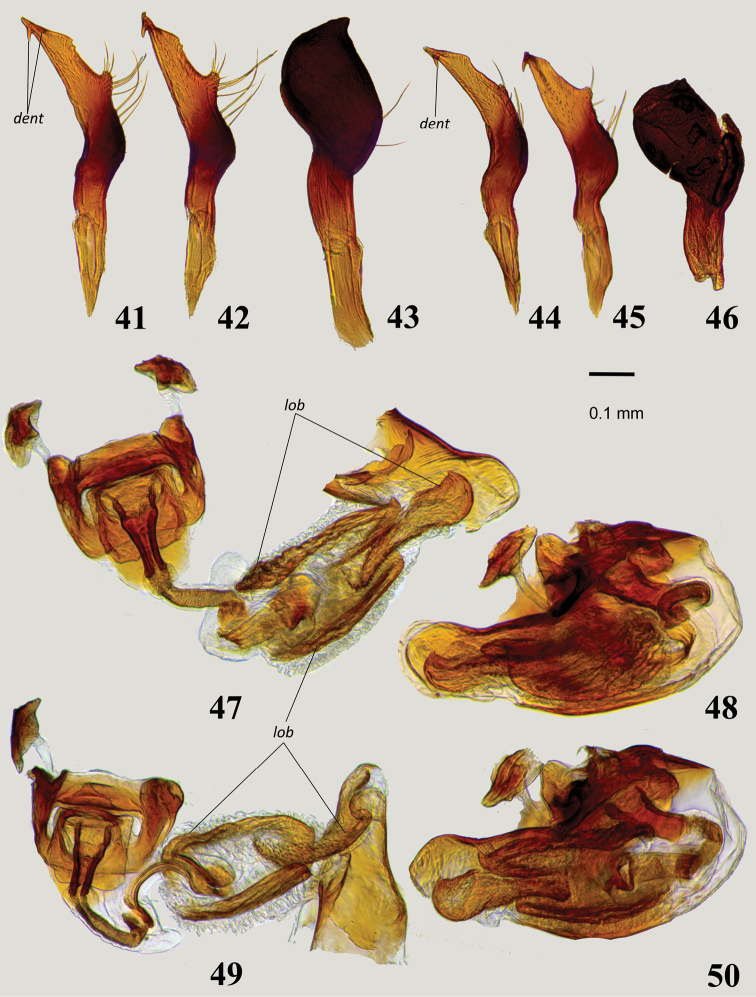
Male genitalia: **41–42, 44–45** Left paramere in dorsal (**41, 44**) and lateral (**42, 45**) views **41, 42***Myrmecophyesnasutus* Drapolyuk, 1989 **44, 45***Myrmecophyestomi* sp. n. **43, 46** Right paramere in dorsal view **43***M.nasutus***46***M.tomi* sp. n. **47–50** Aedeagus in lateral view (**48, 50**) and with phallotheca detached from phallobase and partially expanded endosoma (**47, 49**) **47, 48***M.nasutus*, **49, 50***M.tomi* sp. n. Abbreviations: *dent* –recurved denticle(s) of the left paramere, *lob* – slightly sclerotized lobes of endosoma.

**Figures 51–54.**
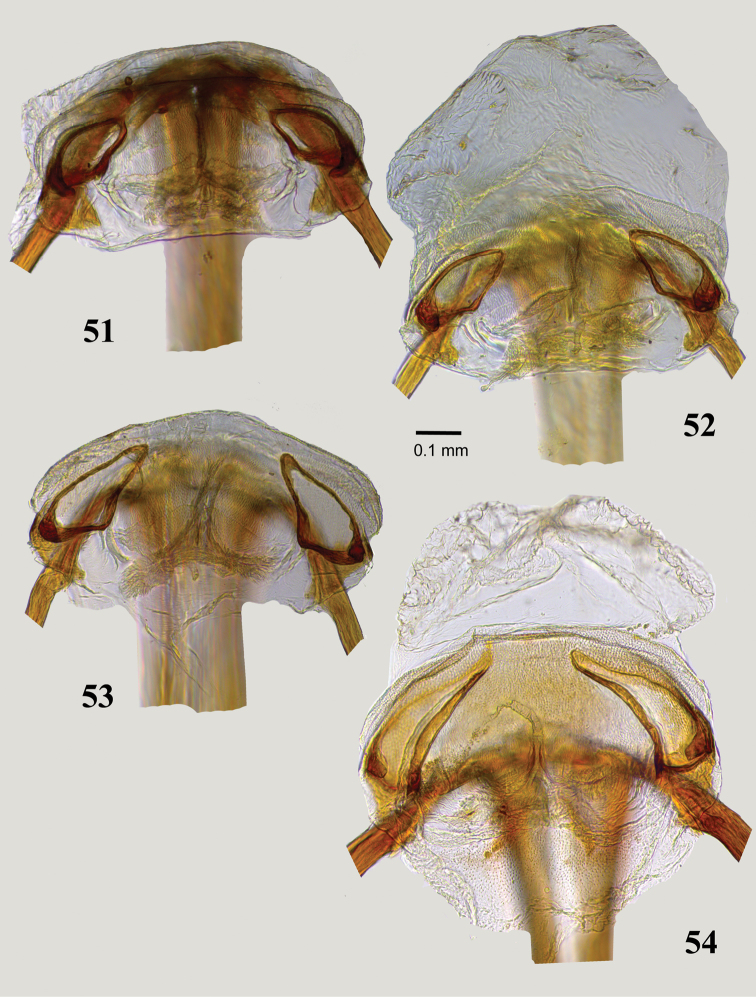
Dorsal labiate plate of bursa copulatrix: **51***Myrmecophyesarmeniacus* Drapolyuk, 1989, **52***Myrmecophyestomi* sp. n. **53***Myrmecophyesheterocerus* Horváth, 1927 **54***Myrmecophyesnasutus* Drapolyuk, 1989.

**Figures 55–63.**
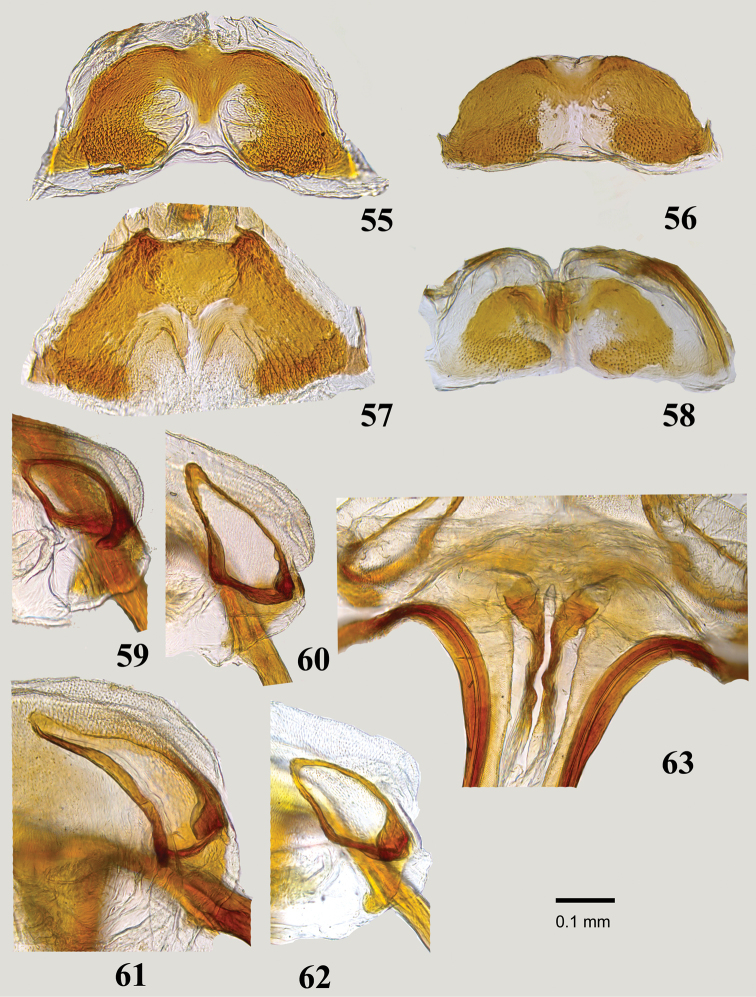
Female genitalia: **55–58** Posterior wall of bursa copulatrix **55***Myrmecophyesarmeniacus* Drapolyuk, 1989 **56***Myrmecophyesheterocerus* Horváth, 1927 **57***Myrmecophyesnasutus* Drapolyuk, 1989 **58***Myrmecophyestomi* sp. n. **59–62** Sclerotized rings of dorsal labiate plate **59***M.armeniacus***60***M.heterocerus***61***Myrmecophyesnasutus***62***M.tomi* sp. n. **63** Vulva of *M.heterocerus*.

**Figure 64.**
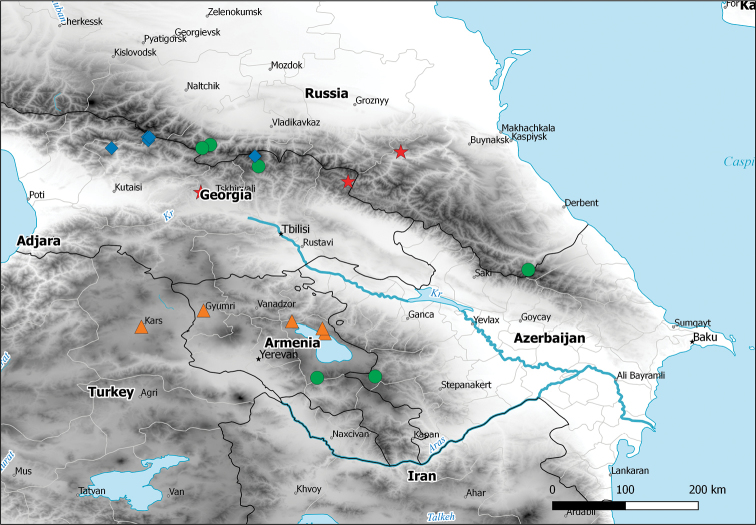
Distribution map of Myrmecophyes (Plumiger) species. Key: ▲ *Myrmecophyesarmeniacus* Drapolyuk, 1989, ● *Myrmecophyesheterocerus* Horváth, 1927, ■ *Myrmecophyesnasutus* Drapolyuk, 1989, ★ *Myrmecophyestomi* sp. n.

## Supplementary Material

XML Treatment for Subgenus Plumiger

XML Treatment for Myrmecophyes armeniacus

XML Treatment for Myrmecophyes heterocerus

XML Treatment for Myrmecophyes nasutus

XML Treatment for Myrmecophyes tomi

## Appendix 1

**Table:** **USI numbers of figured specimens.**

Figure	Species	Sex	USI number
5	* Myrmecophyes armeniacus *	male	AMNH_PBI 00343298
6	* Myrmecophyes heterocerus *	male	AMNH_PBI 00343084
7	* Myrmecophyes tomi *	male	AMNH_PBI 00261443
8	* Myrmecophyes armeniacus *	female	AMNH_PBI 00343318
9	* Myrmecophyes heterocerus *	female	AMNH_PBI 00343142
10	* Myrmecophyes tomi *	female	AMNH_PBI 00261684
11	* Myrmecophyes nasutus *	male	AMNH_PBI 00261441
12	* Myrmecophyes nasutus *	female	AMNH_PBI 00261674
13	* Myrmecophyes nasutus *	female	AMNH_PBI 00261640
14, 16, 22, 23, 25	* Myrmecophyes armeniacus *	female	AMNH_PBI 00343376
15, 17–21, 24	* Myrmecophyes heterocerus *	male	AMNH_PBI 00343378
26	* Myrmecophyes armeniacus *	male	AMNH_PBI 00343291
27	* Myrmecophyes nasutus *	male	AMNH_PBI 00261441
28	* Myrmecophyes heterocerus *	male	AMNH_PBI 00343083
29	* Myrmecophyes tomi *	male	AMNH_PBI 00261443
30–31	* Myrmecophyes armeniacus *	male	AMNH_PBI 00343331
32–33	* Myrmecophyes armeniacus *	male	AMNH_PBI 00343334
34	* Myrmecophyes heterocerus *	male	AMNH_PBI 00343092
35–36	* Myrmecophyes heterocerus *	male	AMNH_PBI 00343129
37–38	* Myrmecophyes armeniacus *	male	AMNH_PBI 00343268
39	* Myrmecophyes heterocerus *	male	AMNH_PBI 00343329
40	* Myrmecophyes heterocerus *	male	AMNH_PBI 00343092
41–43	* Myrmecophyes nasutus *	male	AMNH_PBI 00261441
44–46	* Myrmecophyes tomi *	male	AMNH_PBI 00261443
47–48	* Myrmecophyes nasutus *	male	AMNH_PBI 00261441
49–50	* Myrmecophyes tomi *	male	AMNH_PBI 00261443
51	* Myrmecophyes armeniacus *	female	AMNH_PBI 00343333
52	* Myrmecophyes tomi *	female	AMNH_PBI 00261684
53	* Myrmecophyes heterocerus *	female	AMNH_PBI 00261637
54	* Myrmecophyes nasutus *	female	AMNH_PBI 00261646
55	* Myrmecophyes armeniacus *	female	AMNH_PBI 00343333
56	* Myrmecophyes heterocerus *	female	AMNH_PBI 00343339
57	* Myrmecophyes nasutus *	female	AMNH_PBI 00261646
58	* Myrmecophyes tomi *	female	AMNH_PBI 00261444
59	* Myrmecophyes armeniacus *	female	AMNH_PBI 00343333
60	* Myrmecophyes heterocerus *	female	AMNH_PBI 00261637
61	* Myrmecophyes nasutus *	female	AMNH_PBI 00261646
62	* Myrmecophyes tomi *	female	AMNH_PBI 00261684
63	* Myrmecophyes heterocerus *	female	AMNH_PBI 00261637
